# Evolutionary Distance of Amino Acid Sequence Orthologs across Macaque Subspecies: Identifying Candidate Genes for SIV Resistance in Chinese Rhesus Macaques

**DOI:** 10.1371/journal.pone.0123624

**Published:** 2015-04-17

**Authors:** Cody T. Ross, Morteza Roodgar, David Glenn Smith

**Affiliations:** 1 Department of Anthropology, University of California, Davis. Davis, United States of America; 2 Molecular Anthropology Laboratory, University of California, Davis. Davis, United States of America; 3 California National Primate Research Center, University of California, Davis. Davis, United States of America; 4 Graduate Group of Comparative Pathology, University of California, Davis. Davis, United States of America; Boston College, UNITED STATES

## Abstract

We use the Reciprocal Smallest Distance (RSD) algorithm to identify amino acid sequence orthologs in the Chinese and Indian rhesus macaque draft sequences and estimate the evolutionary distance between such orthologs. We then use GOanna to map gene function annotations and human gene identifiers to the rhesus macaque amino acid sequences. We conclude methodologically by cross-tabulating a list of amino acid orthologs with large divergence scores with a list of genes known to be involved in SIV or HIV pathogenesis. We find that many of the amino acid sequences with large evolutionary divergence scores, as calculated by the RSD algorithm, have been shown to be related to HIV pathogenesis in previous laboratory studies. Four of the strongest candidate genes for SIV_*mac*_ resistance in Chinese rhesus macaques identified in this study are *CDK*9, *CXCL*12, *TRIM*21, and *TRIM*32. Additionally, *ANKRD*30*A*, *CTSZ*, *GORASP*2, *GTF*2*H*1, *IL*13*RA*1, *MUC*16, *NMDAR*1, *Notch*1, *NT*5*M*, *PDCD*5, *RAD*50, and *TM*9*SF*2 were identified as possible candidates, among others. We failed to find many laboratory experiments contrasting the effects of Indian and Chinese orthologs at these sites on *SIV_mac_* pathogenesis, but future comparative studies might hold fertile ground for research into the biological mechanisms underlying innate resistance to *SIV_mac_* in Chinese rhesus macaques.

## Introduction

Recent work has produced complete genome sequence data for both the Indian [1] and Chinese rhesus macaque [2]. The existence of these draft sequences plays a very crucial role in permitting comparative genomic study of the differentiation of Chinese and Indian rhesus macaque amino acid sequences.

Amino acid sequence divergence between Chinese and Indian rhesus macaques has the potential to influence phenotype, especially response to immune system challenge, as genes related to immune response are some of the most rapidly evolving across species [3–6]. Additionally, evolution of the regulatory regions of genes is also known to play a very significant role in immunity [7, 8]. Thus, heterogeneity in phenotypic response to pathogens is a function not only of what protein is expressed by a gene, but also a function of the timing [9, 10], tissue type/biological location [11], quantity [12], and interaction [12] of genes being expressed.

It has been shown that Chinese rhesus macaques generally have increased elite controller status and more frequent long-term non-progression to simian-AIDs relative to Indian rhesus macaques [13, 14]. Understanding the genetic sources of heterogeneity in immune response to SIV_*mac*_ between Chinese and Indian rhesus macaques may thus provide important insights into SIV_*mac*_ and HIV/AIDS biology more generally.

In this study, we focus on identifying genes related to HIV or SIV_*mac*_ whose amino acid sequences show high levels of divergence across Chinese and Indian rhesus macaques, as these genes may be strong candidates for the difference in immunity to SIV_*mac*_ across these subspecies. We do not investigate divergence in non-coding regulatory regions, although we believe such divergence to play a significant role in heterogeneity in immune response across subspecies. Including data from non-coding regions in our analysis would create significant methodological and computational difficulties at this point in time, but we believe that our methods will be amenable to such investigations as computational methods increase in power and efficiency, and genomic data increases in quantity and quality.

Previous comparative genomic investigations for evidence of selection [2] and high throughput single-nucleotide polymorphism (SNP) sequencing and linkage disequilibrium analysis [13] in Chinese and Indian rhesus macaques have found patterns consistent with positive selection on genes such as *TCTE*1, *ZNF*337, and *TRIM*5 [2], and *MAST*2, *ELAVL*4, and *HIVEP*3 [13]. The functional linkages between the gene candidates presented in these papers and resistance to SIV_*mac*_ in Chinese rhesus macaques still remain ambiguous, however, as further laboratory work is needed to investigate the relevance of Chinese versus Indian orthologs on SIV_*mac*_ pathogenesis.

In this analysis, we attempt to identify additional candidate genes for SIV_*mac*_ resistance and test if we can re-identify previously described candidate genes using a new methodology. Our methods are similar at first pass to those used by Yan et al [2], in that we attempt to identify orthologous amino acid sequences between Chinese and Indian rhesus macaques using complete draft sequence data and use synteny mapping to evaluate the performance of the ortholog classifications. Our methods differ in that we do not infer orthology on the basis of synteny, but instead use a Python implementation of the Reciprocal Smallest Distance (RSD) algorithm [15] to evaluate orthology and then use synteny mapping to check the performance of the RSD algorithm.

The RSD algorithm functions to detect putative orthologs using sequence alignment in a way similar to the reciprocal best hit algorithm (RBH) [16]. The RSD algorithm, however, works around a shortcoming of the RBH algorithm that occurs when a forward blast yields a paralog best hit, but a reciprocal blast recovers an ortholog; in such cases, the RBH alogorithm excludes both pairs, but the RSD algorithm can often recover the true ortholog [15]. The RSD algorithm accomplishes this by conducting a forward blast of an amino acid sequence, *i*, from a draft sequence, *I*, onto a secondary draft sequence, *J*, to obtain a list of hits, *H*. Instead of simply checking for reciprocal best hits, the RSD algorithm moves on to compare each hit, *h*, meeting threshold criteria against the query sequence, *i*. A maximum likelihood estimate, *e*, of the evolutionary distance (substitutions per site) separating *h* and *i* is calculated, given an empirical amino acid substitution rate matrix [15]. Note that these numbers might appear surprisingly large if the focal amino acid sequences are of different lengths. For instance, the inferred orthologous amino acid sequences ENSMMUP00000038625 and ENSP00000407071 (GO identifier RAD50) are of very different lengths, increasing the associated *e* value to 2.2, even though the percent identify matrix from Clustal 2.1 shows a small but plausible sequence identity of 39.47 percent. Of all considered sequences in *H*, only the sequence with the smallest evolutionary distance is retained; this sequence, *j*, is then used for a reciprocal blast against the first draft sequence, *I* [15]. Hits from this blast are treated analogously, and an orthologous pair is considered to be found if and only if sequences *i* and *j* are the sequences with reciprocal smallest evolutionary distances [15].

An attractive feature of the RSD algorithm is that it provides estimates of evolutionary distance [17] between each pair of othologs. Given that the Indian and Chinese rhesus macaque sub-populations are estimated to have separated only about 160,000 years ago [18], a large majority of the genome is expected to be relatively constant across sub-populations. Thus, large differences in amino acid sequence between orthologous pairs in Chinese and Indian rhesus macaques are notable. Given that bacteria or viruses with structural, functional, or morphological similarities to *SIV*_*mac*_ stand to place selective pressure on genes which might confer resistance or even elite controller status to such infections, and Chinese and Indian rhesus macaques show different normative phenotypes in relation to *SIV*_*mac*_ pathogenesis, orthologs with negligible (∼ *e* < 0.05) evolutionary distance (about 15,448 of the 17,064 amino acid sequences considered in this study) across Chinese and Indian rhesus macaques are unlikely to be responsible for differences in resistance to *SIV*_*mac*_, conditional on the assumption that coding region variation is the key driver of this phenotypic difference. Orthologs with non-negligible evolutionary distance across Chinese and Indian rhesus macaques are candidates for genes that might be diverging due to a disjunctive array of demographic and selection pressures, of which immune system challenge is only a single example.

To narrow down which of these amino acid sequences might contribute to SIV_*mac*_ resistance in Chinese rhesus macaques, we used GOanna [19] to associate gene symbols (human homologs) and gene function annotations to each rhesus macaque amino acid sequence, and then cross-tabulated the portion of our candidate list with the highest divergence/evolutionary distance scores (492 genes in total with divergence scores *e* > 0.15) with a list of 140 genes known to be involved in HIV-1 pathogenesis from a thorough literature review [20], a list of 169 candidate genes for HIV-1 susceptibility derived from a large-scale genome-wide common-disease common-variant analysis of HIV-1 in Africa [21], and four genome-wide siRNA analyses [22–25].

Additionally, we conducted a literature review of the 25 orthologs with the largest divergence/evolutionary distance scores from our RSD analysis in order to investigate if any of these genes had been associated with HIV or SIV pathogenesis by laboratory studies not cited in the previous literature reviews or association studies [20].

We complete our analysis by: 1) modeling ortholog divergence scores as a function of chromosome and chromosomal location (on the Indian rhesus macaque reference frame) to investigate if there are any distinct chromosomal locations with high concentrations of divergent orthologs, and 2) investigating divergence scores as a function of gene ontology classification categories.

While many disjunctive ecological differences may have led to divergent amino acid sequence evolution between Chinese and Indian rhesus macaques, researchers are especially interested in understanding how divergent evolution in each sub-population has led one sub-population (Chinese rhesus macaques) to become resistant to the simian analog of HIV [13, 14]. While careful laboratory studies are required to uncover the role of specific genes (and their interactions) on SIV_*mac*_ and HIV resistance, wide-scale comparative genomic scans may provide useful insights and substantially reduce the list of candidate genes to be investigated with more rigorous laboratory methods; uncovering the functional and biological significance of genetic variation is made easier by integrating the partial strengths of genomic, evolutionary, and biochemical data and methods [26].

## Results

### Establishing Orthology between Chinese and Indian Rhesus Macaque Reference Sequences

Supplementary S1 Table includes the full list of Chinese and Indian amino acid sequence orthologs, paired with the location information of each ortholog on each draft sequence, the estimated divergence score from the RSD algorithm, and the associated gene symbols from GOanna.

**Fig 1:**
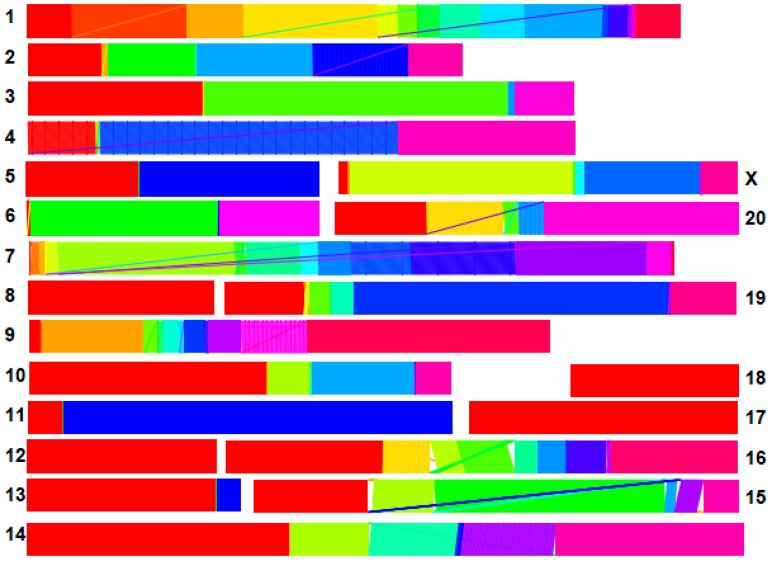
Synteny maps between Chinese and Indian rhesus macaque reference sequences. The large-scale concordance serves to verify that the RSD algorithm is functioning to accurately map orthologous sequences. Each colored line maps an amino acid sequence in the Indian rhesus macaque draft sequence (top) to its corresponding ortholog on the Chinese rhesus macaque draft sequence (bottom). Diagonal lines illustrate transpositions; thin diagonal lines are indicative of single amino acid transposition, and thicker diagonal lines are indicative of block transpositions. The differing colors on each chromosome serve to illustrate the blocks in which synteny is fully conserved. Most chromosomes show almost fully conserved synteny, but chromosomes 15 and 16 show fairly large-scale rearrangement.

Fig 1 plots the synteny maps implied by the ortholog classification of the RSD algorithm, dropping a small number of amino acid sequences that the RSD algorithm believes to have undergone inter-chromosomal transposition. These maps show that the vast majority of orthologous pairs are contained in expansive blocks of synteny. We observe only a small number of apparent intra-chromosomal transpositions per chromosome, the majority of which are inversions of neighboring amino acid sequences. On chromosomes 15, 16, and 19 we observed small intra-chromosomal block transpositions.

### Identifying the Orthologs with Highest Divergence between Chinese and Indian Rhesus Macaques

We cross-tabulated our candidate list of 492 genes with divergence values of *e* > 0.15 with several lists of genes (Supplementary S2 Table) suspected to be involved in HIV or SIV pathogenesis from thorough literature review [20], association studies [21], or genome-wide siRNA analyses [22–25].

Using this methodology we identified five genes (*CDK*9, *CXCL*12, *LPL*, *TRIM*32, and *TRIM*21) from the set of genes described by [20], five genes (*CXCL*12, *CYP*26*B*1, *EFR*3*A*, *NME*6, and *PIGX*) from the set of genes provided by [21], zero genes from the set of gene provided by [22], four genes (*BCAR*1, *DSP*, *ICAM*4, and *SLC*35*B*1) from the set of gene provided by [23], six genes (*DAPK*2, *PARVA*, *CTSZ*, *GTF*2*H*1, *LYPLA*1, and *GORASP*2) from the set of gene provided by [24], and three genes (*ANKRD*30*A*, *LPL*, and *TM*9*SF*2) from the set of genes provided by [25]. These twenty genes are listed in Table 1 with their divergence scores and identifiers on the Indian and Chinese rhesus macaque reference frames.

**Table 1:** Table 1 presents the 20 genes that have been linked to HIV pathogenesis in previous studies [20–25] and also have highly divergent orthologs across Chinese and Indian rhesus macaques. The label “GOname” indicates the gene name assigned by GOanna. The labels “ProteinID.IR” and “ProteinID.CR” indicate the name of the amino acid sequence in the Indian and Chinese rhesus macaque draft sequence files, respectively. The label “*e*” is the estimate of evolutionary distance produced by the RSD alogirthm via implentation of PAML [17]. Finally, the labels “Chr.IR” and “Chr.CR” indicate the chromosome on which the amino acid sequences occur in the Indian and Chinese rhesus macaque draft sequence files, respectively.

**GOname**	**ProteinID.IR**	**ProteinID.CR**	***e***	**Chr.IR**	**Chr.CR**	**HIV-Related**	**Reference**
TRIM32	ENSMMUP00000017213	ENSP00000345464	2.4021	15	4	✓	[20]
ICAM4	ENSMMUP00000012237	ENSP00000342114	0.7103	19	19	✓	[22, 23]
BCAR1	ENSMMUP00000032314	ENSP00000391669	0.51	20	20	✓	[23]
TRIM21	ENSP00000339355	ENSP00000381338	0.4936	14	14	✓	[20]
CXCL12	ENSMMUP00000035953	ENSP00000379140	0.4456	9	9	✓	[20, 21]
DAPK2	ENSMMUP00000023902	ENSP00000408277	0.4175	7	7	✓	[24]
CTSZ	ENSMMUP00000020183	ENSP00000217131	0.3892	10	10	✓	[24]
PARVA	ENSMMUP00000027577	ENSP00000334008	0.2632	14	14	✓	[24]
GORASP2	ENSMMUP00000020692	ENSMMUP00000002335	0.2488	16	16	✓	[24]
EFR3A	ENSMMUP00000016487	ENSMMUP00000016487	0.2401	8	8	✓	[21]
PIGX	ENSMMUP00000005029	ENSP00000296333	0.2396	2	2	✓	[21]
DSP	ENSMMUP00000038586	ENSP00000400669	0.2286	11	11	✓	[23]
TM9SF2	ENSMMUP00000025694	ENSP00000365567	0.196	17	17	✓	[25]
GTF2H1	ENSMMUP00000041182	ENSMMUP00000023608	0.1926	18	14	✓	[24]
NME6	ENSMMUP00000004110	ENSP00000416658	0.1853	2	2	✓	[21]
LPL	ENSMMUP00000006264	ENSP00000309757	0.184	8	8	✓	[20, 25]
ANKRD30A	ENSMMUP00000005876	ENSP00000386398	0.1816	12	12	✓	[25]
CYP26B1	ENSMMUP00000015927	ENSP00000001146	0.1762	13	13	✓	[21]
LYPLA1	ENSMMUP00000017285	ENSP00000397807	0.1729	8	5	✓	[24]
SLC35B1	ENSMMUP00000034903	ENSP00000409548	0.1599	16	16	✓	[23]
CDK9	ENSMMUP00000017333	ENSP00000362362	0.1504	15	15	✓	[20]

Additionally, we conducted our own literature review of the 20 genes with the largest divergence scores from our RSD analysis; Table 2 presents our results. We find that at least 7 of the 20 genes with the highest divergence between the Chinese and Indian orthologs have been linked to HIV or SIV pathogenesis in some way by previous studies.

**Table 2:** Table 2 presents the 20 orthologs with the highest levels of divergence according to the RSD algorithm. Of these 20 genes, 7 have been linked to HIV-1 pathogenesis in some way by previous research. The label “GOname” indicates the gene name assigned by GOanna. The labels “ProteinID.IR” and “ProteinID.CR” indicate the name of the amino acid sequence in the Indian and Chinese rhesus macaque draft sequence files, respectively. The label “*e*” is the estimate of evolutionary distance produced by the RSD alogirthm via implentation of PAML [17]. Finally, the labels “Chr.IR” and “Chr.CR” indicate the chromosome on which the amino acid sequences occur in the Indian and Chinese rhesus macaque draft sequence files, respectively.

**GOname**	**ProteinID.IR**	**ProteinID.CR**	***e***	**Chr.IR**	**Chr.CR**	**HIV Related**	**Reference**
TRIM32	ENSMMUP00000017213	ENSP00000345464	2.4021	15	4	✓	[33, 38, 41, 88, 89]
RAD50	ENSMMUP00000038625	ENSP00000407071	2.2032	2	2	✓	[65]
KLHL42	ENSP00000321544	ENSP00000379034	1.4254	8	10	-	-
NMDAR1	ENSMMUP00000009665	ENSP00000381630	1.3902	8	8	✓	[66, 90, 91]
Adcy10	ENSMMUP00000027891	ENSP00000410352	1.2541	4	4	-	-
MUC16	ENSMMUP00000020314	ENSP00000381008	1.1982	19	19	✓	[69, 70]
BCMO1	ENSMMUP00000000974	ENSP00000258168	1.1973	20	20	-	-
IL13RA1	ENSMMUP00000015103	ENSP00000360730	1.1112	X	X	✓	[74]
NT5M	ENSMMUP00000035155	ENSP00000390695	1.0756	16	16	✓	[75]
Prss27	ENSMMUP00000020977	ENSP00000161006	1.0391	20	20	-	-
YY2	ENSMMUP00000029555	ENSP00000368798	1.0059	X	X	-	-
Sec22b	ENSMMUP00000015200	ENSP00000362504	0.9357	4	4	?	[92, 93]
FAM110A	ENSP00000363386	ENSP00000363386	0.9143	1	1	?	[94]
Notch1	ENSMMUP00000036150	ENSP00000398674	0.901	13	13	✓	[76, 95]
RBM38	ENSMMUP00000038172	ENSMMUP00000014941	0.887	10	4	-	-
GRIN3A	ENSMMUP00000021930	ENSP00000234389	0.8687	15	19	-	-
CCDC176	ENSMMUP00000016656	ENSP00000377577	0.817	7	7	-	-
FBXO45	ENSMMUP00000038210	ENSP00000310332	0.8117	2	2	-	-
FRS2	ENSMMUP00000039208	ENSP00000381083	0.8032	11	11	-	-
PDCD5	ENSMMUP00000020187	ENSP00000388543	0.7491	19	19	✓	[78]

### Identifying Evolutionary Divergence between Chinese and Indian Rhesus Macaque Orthologs as a Function of Chromosomal Region

**Fig 2:**
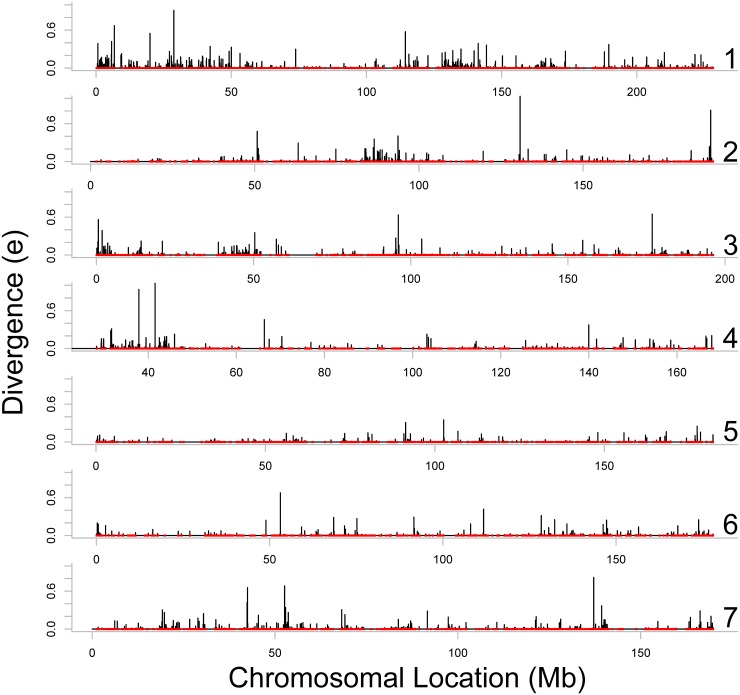
Evolutionary distance between Indian and Chinese rhesus macaque orthologs, localized on the Indian rhesus macaque draft sequence (Chromosomes 1 through 7). The red points illustrate the locations of the orthologs (including the orthologs with *e* = 0) on the horizontal axis, and the black lines represent the divergence scores (*e*) of these orthologs on the vertical axis. The area of elevated evolutionary distance on the beggining portion of Chromosome 1 corresponds to an area with significant differences in linkage disequilibrium between Indian-derived and Chinese rhesus macaques [13].

**Fig 3:**
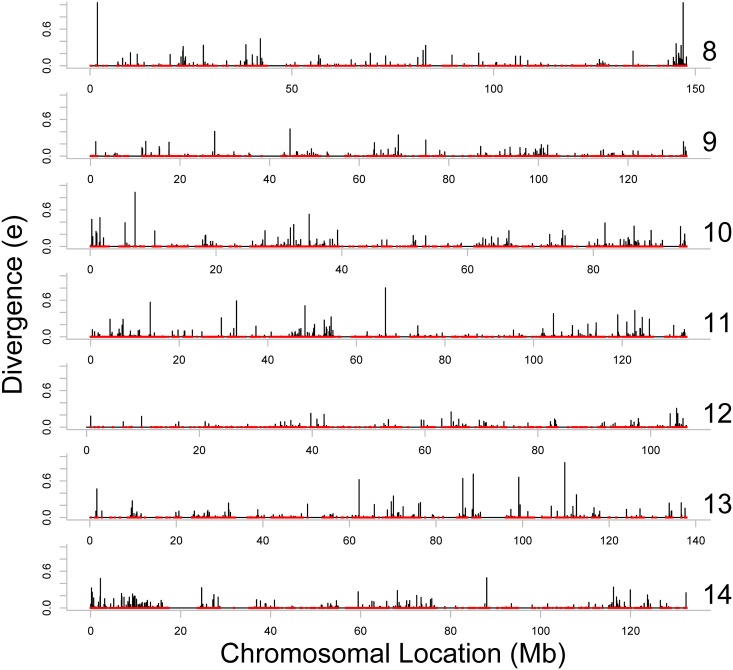
Evolutionary distance between Indian and Chinese rhesus macaque orthologs, localized on the Indian rhesus macaque draft sequence (Chromosomes 8 through 14). The red points illustrate the locations of the orthologs (including the orthologs with *e* = 0) on the horizontal axis, and the black lines represent the divergence scores (*e*) of these orthologs on the vertical axis. The end region of chromosome 8 shows an area of high divergence.

**Fig 4:**
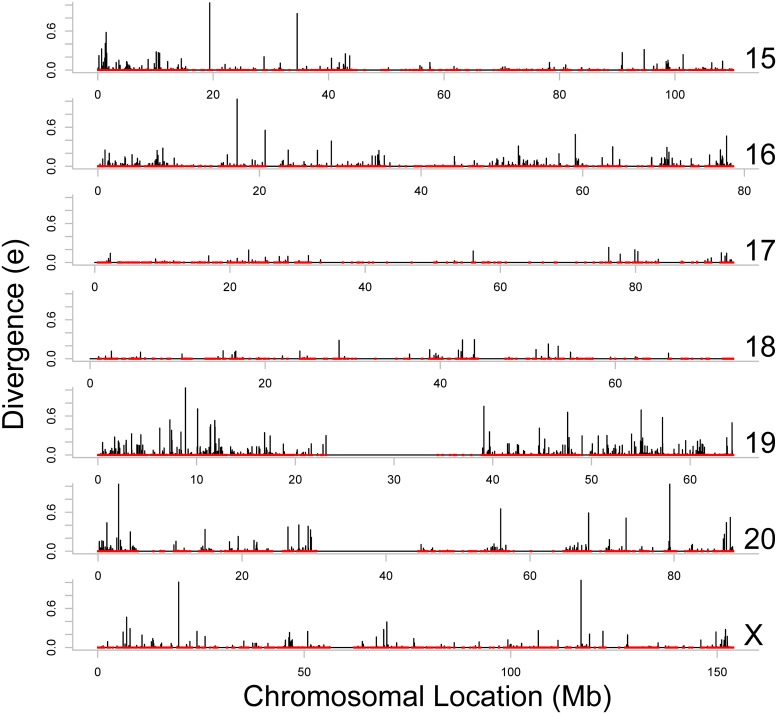
Evolutionary distance between Indian and Chinese rhesus macaque orthologs, localized on the Indian rhesus macaque draft sequence (Chromosomes 15 through 20, and X). The red points illustrate the locations of the orthologs (including the orthologs with *e* = 0) on the horizontal axis, and the black lines represent the divergence scores (*e*) of these orthologs on the vertical axis. Chromosome 19 shows consistently high evolutionary divergence.

**Fig 5:**
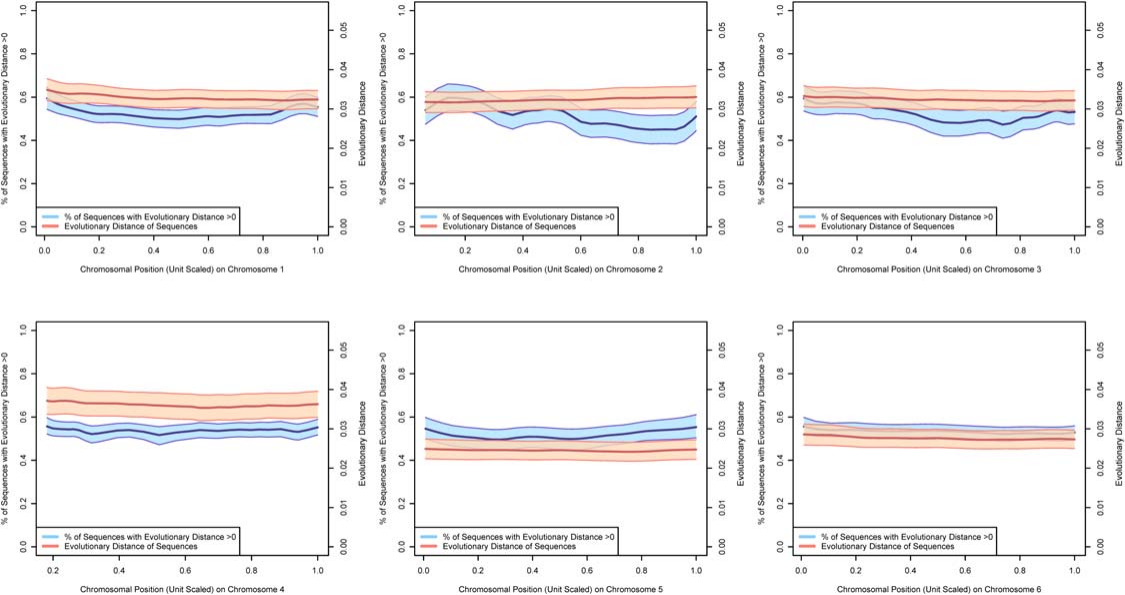
The 70% posterior credibility intervals from the zero-inflated gamma regression model for chromosomes 1–6. The blue confidence band plots the mean probability that an amino acid sequence is divergent as a function of chromosomal position on the left axis and the orange confidence band plots the mean evolutionary distance of the subset of diverging amino acid sequences as a function of chromosomal position on the right axis. Across all chromosomes, we note little difference in divergence of amino acid sequences as a function of chromosomal position, but some differences in mean evolutionary divergence across chromosomes.

**Fig 6:**
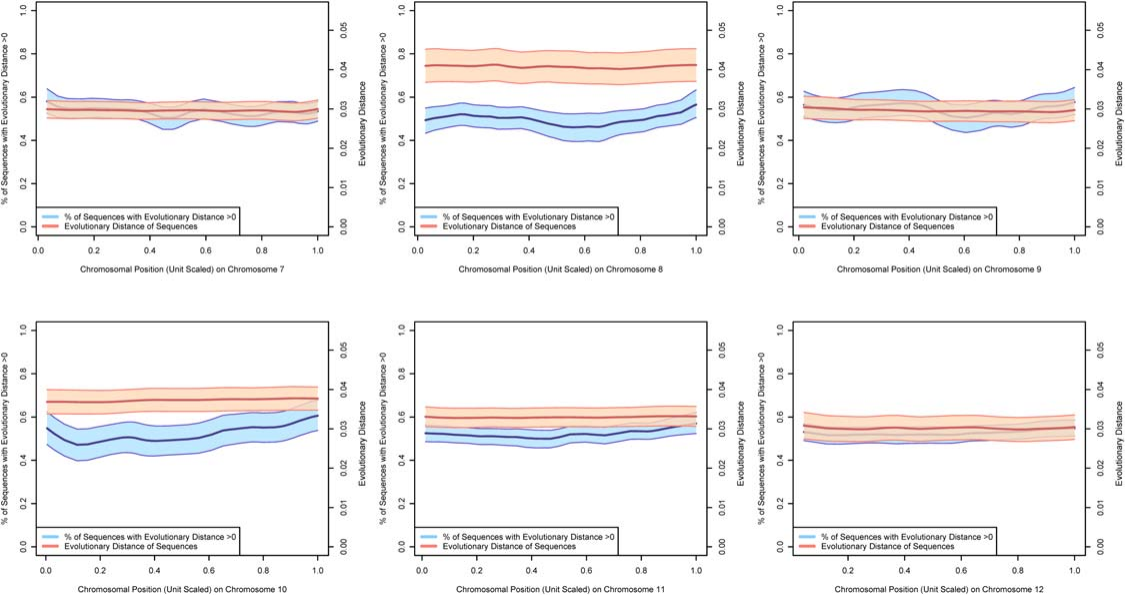
The 70% posterior credibility intervals from the zero-inflated gamma regression model for chromosomes 7–12. The blue confidence band plots the mean probability that an amino acid sequence is divergent as a function of chromosomal position on the left axis and the orange confidence band plots the mean evolutionary distance of the subset of diverging amino acid sequences as a function of chromosomal position on the right axis. Across all chromosomes, we note little difference in divergence of amino acid sequences as a function of chromosomal position, but some differences in mean evolutionary divergence across chromosomes.

**Fig 7:**
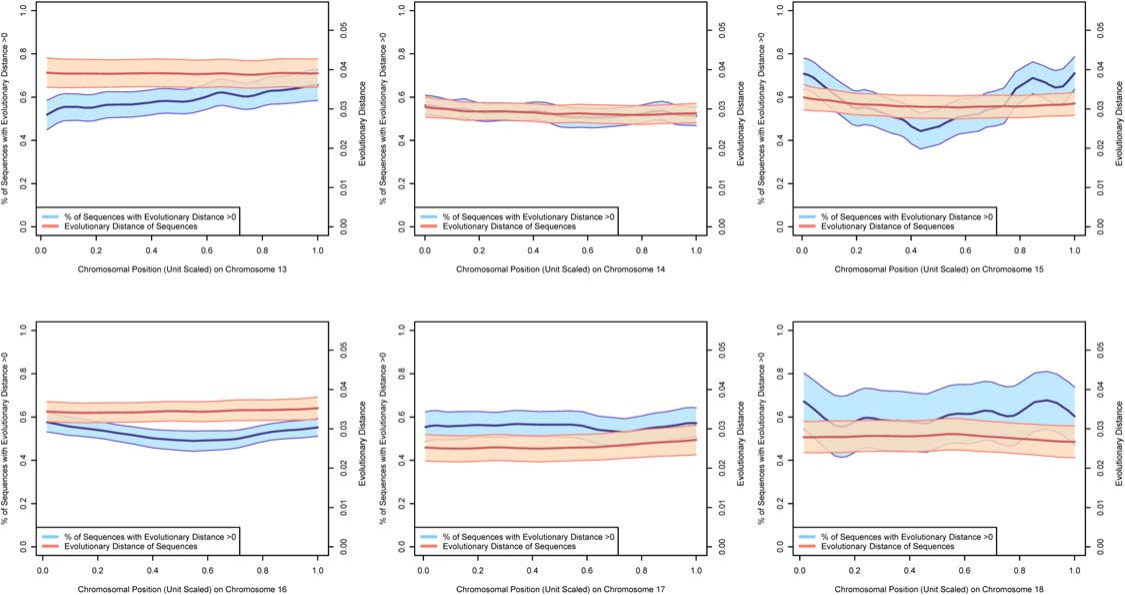
The 70% posterior credibility intervals from the zero-inflated gamma regression model for chromosomes 13–18. The blue confidence band plots the mean probability that an amino acid sequence is divergent as a function of chromosomal position on the left axis and the orange confidence band plots the mean evolutionary distance of the subset of diverging amino acid sequences as a function of chromosomal position on the right axis. Across all chromosomes, we note little difference in divergence of amino acid sequences as a function of chromosomal position, but some differences in mean evolutionary divergence across chromosomes.

**Fig 8:**
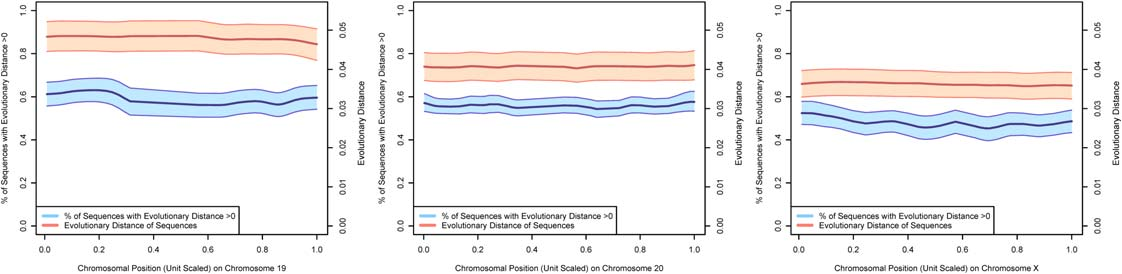
The 70% posterior credibility intervals from the zero-inflated gamma regression model for chromosomes 19–20 and X. The blue confidence band plots the mean probability that an amino acid sequence is divergent as a function of chromosomal position on the left axis and the orange confidence band plots the mean evolutionary distance of the subset of diverging amino acid sequences as a function of chromosomal position on the right axis. Across all chromosomes, we note little difference in divergence of amino acid sequences as a function of chromosomal position, but some differences in mean evolutionary divergence across chromosomes.

Figs 2–4 present estimates of evolutionary distance between the Chinese and Indian amino acid sequences as a function of chromosomal location in megabases (Mb). Amino acid sequence divergence levels appear to show weak signs of concentration into regions on a chromosome, although the extent to which this pattern holds may be confounded by the clustering of amino acid sequences themselves into specific regions on each chromosome. To better estimate chromosomal position effects, we use a multi-level, zero-inflated gamma regression model, with random effects on chromosomal position generated using a Gaussian process. Across chromosomes, we see very little evidence for strong chromosomal position effects, as there is very little difference in the estimated probability of a non-zero evolutionary distance or the mean value of evolutionary distance as a function of chromosomal position. These results are plotted in Figs 5–8.

**Fig 9:**
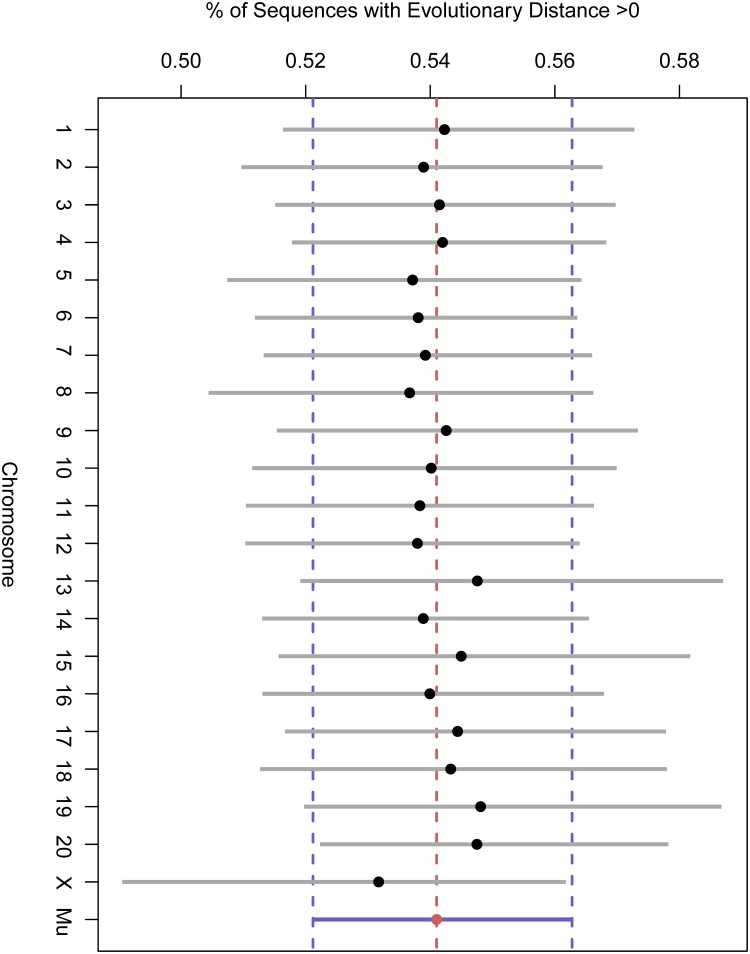
The 90% posterior credibility intervals of the chromosomal random effects from the Bernoulli sub-model of the zero-inflated gamma regression. The value ‘Mu’ is the partially-pooled mean estimate across chromosomes. We see that there are no significant differences in the mean probabilty of non-zero divergence in amino acid sequences across chromosomes.

**Fig 10:**
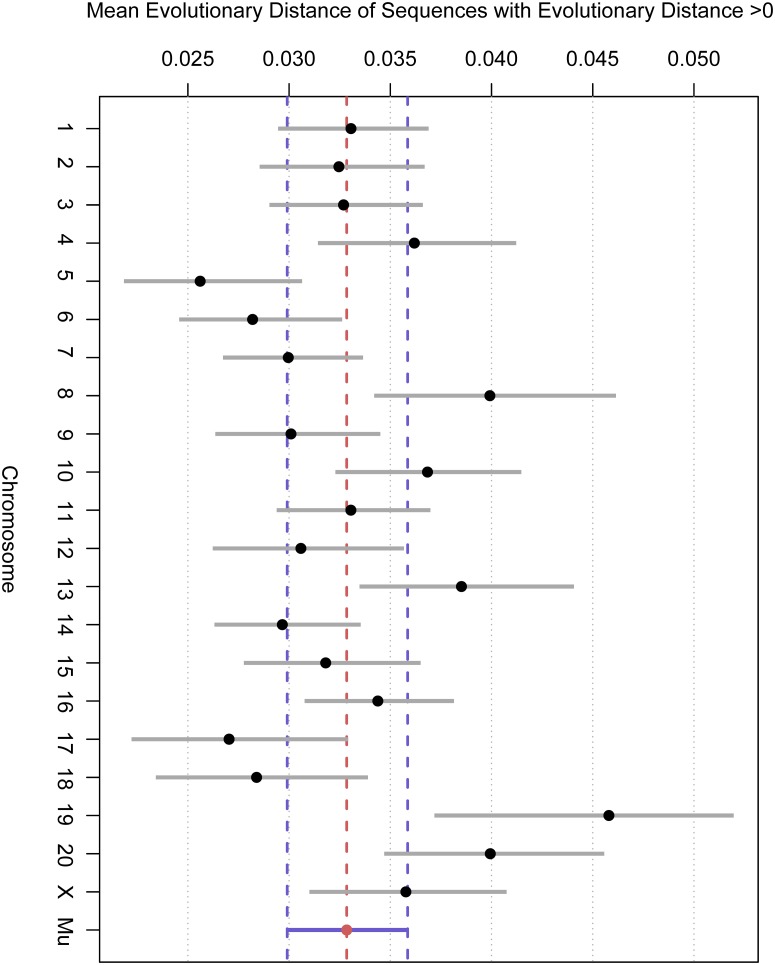
The 90% posterior credibility intervals of the chromosomal random effects from the gamma sub-model of the zero-inflated gamma regression. The value ‘Mu’ is the partially-pooled mean estimate across chromosomes. We see that there is fairly significant heterogeneity in mean evolutionary divergence of amino acid sequences across chromosomes, with chromosomes 5, 6, and 17 showing reduced evolutionary divergence, and chromosomes 8, 13, 19, and 20 showing increased evolutionary divergence.

We did observe significant heterogeneity in mean evolutionary distance across chromosomes, with some (e.g. 5, 6, and 17) showing reduced amino acid sequence divergence and others (e.g. 8, 13, 19, and 20) showing increased evolutionary divergence. These results are plotted in Figs 9 and 10.

### Identifying the Gene Function Classes with Highest Evolutionary Divergence between Chinese and Indian Rhesus Macaques

To investigate the functional roles of genes associated with increased ortholog divergence scores, we used GOanna to link gene annotations with ortholog pairs. Table 3 presents the 20 gene function classes (of the 176 containing at least 100 genes) with highest median divergence scores across the rhesus macaque genome. Supplementary S3 Table contains the complete annotation list paired with ortholog divergence scores. Genes involved in viral processes and immunity show elevated levels of evolutionary divergence.

**Table 3:** Table 3 presents the 20 gene ontology (GO) categories (of the 176 GO categories containing at least 100 genes) with the highest median levels of divergence according to the RSD algorithm. The label “Zeros” refers to the number of genes in the GO category with a divergence score of zero. The label “N” refers to the number of genes in the GO category. GO categories related to viral processes and immunity are well represented among the GO categories that are diverging at higher rates relative to most other categories. We note that 3 of the 20 (15%) most divergent GO categories are related to viral processes or immunity, while only 2 of the other 156 (1%) of GO categories are related to viral processes or immunity. Table 3 is a subset of Supplementary S3 Table.

GO Category	**Mean**	**Median**	**Max**	**SD**	**Zeros**	**N**
translational termination	0.012	0.006	0.268	0.027	90	214
**viral transcription**	0.012	0.006	0.268	0.027	90	214
translational elongation	0.012	0.006	0.268	0.026	93	218
**viral life cycle**	0.012	0.006	0.268	0.027	95	220
nuclear-transcribed mRNA catabolic process, nonsense-mediated decay	0.012	0.005	0.268	0.025	106	241
translational initiation	0.011	0.005	0.268	0.025	103	236
SRP-dependent cotranslational protein targeting to membrane	0.013	0.005	0.29	0.032	107	238
cytosolic large ribosomal subunit	0.011	0.005	0.177	0.023	63	133
protein complex binding	0.024	0.004	0.407	0.054	37	111
**viral process**	0.013	0.004	0.268	0.029	181	405
anatomical structure morphogenesis	0.017	0.003	0.264	0.043	44	108
detection of chemical stimulus involved in sensory perception of smell	0.008	0.003	0.091	0.013	53	152
olfactory receptor activity	0.008	0.003	0.091	0.013	53	152
sensory perception of smell	0.008	0.003	0.091	0.014	67	176
actin cytoskeleton	0.021	0.003	0.405	0.054	53	139
endoplasmic reticulum lumen	0.025	0.003	0.481	0.061	35	108
oxidation-reduction process	0.033	0.003	1.197	0.128	41	120
cytoskeleton organization	0.012	0.003	0.192	0.028	37	108
**cell surface**	0.023	0.003	0.337	0.053	93	268
**external side of plasma membrane**	0.023	0.003	0.446	0.058	47	129

## Discussion

### RSD Performance

The RSD algorithm appears to function with high accuracy in identifying orthologs, as synteny maps show large blocks of gene order conservation, sometimes spanning the entire chromosome (e.g. on chromosomes 8, 12, 17, and 18). There is, however, evidence of broken synteny on some chromosomes due to single amino acid sequence transpositions (e.g. on chromosomes 1, 4, 7, 9 and 20), as well as block transpositions (e.g. on chromosomes 14, 15, 16, and 19). Additionally, RSD believed some amino acid sequences to have orthologs on different chromosomes.

Some researchers [2] opt to exclude proposed orthologs if the pair break blocks of synteny. We have not excluded any potential orthologs from our results based on synteny breaking, but we have marked (in Supplementary S1 Table) which potential orthologs break synteny or occur on different chromosomes (387 of 17,064 proposed orthologs showed such behavior). It is possible that potential orthologs which break synteny are either 1) mis-classified as orthologs by RSD, or 2) poorly localized in the draft sequences. If (1) is the case, then our estimates of evolutionary distance are invalid; if (2) is the case, then our estimates of evolutionary distance are valid, but our localizations are invalid.

We, however, find it possible that many of the transpositions discovered by RSD are true transpositions. RSD uses global information and minimum distance criteria to map orthologs, so misclassifications are unlikely conditional on the global information being complete, amino acid sequences being large, and divergence times being small. Transpositions (both intra- and inter-chromosomal) and small inversions are known to occur during the evolutionary process, and are frequently seen in inter-species mappings [27]. It is extremely unlikely to see transposition of blocks in which synteny is conserved if the sequences in the transposed blocks are not true orthologs. In such cases, however, the appearance of transpositions or inversions may be due to errors in localization of orthologs on one or both of the reference sequences.

Transpositions may be indicative of important inter-gene interactions due to chromosomal position effects [28], epigenetic effects [29], or simply lowered odds of recombination separating alleles that have non-additive or synergistic effects (see Maynard-Smith’s argument for the evolutionary origin of chromosomes [30, 31]). By presenting the data and results without exclusion, we leave it to the reader to interpret the findings, while acknowledging where possible confounding methodological factors may inhibit strong inference.

### Candidate Genes

We provide a brief and by no means exhaustive review of laboratory studies that address the functional relationships between the candidate genes for SIV_*mac*_ resistance uncovered in this study and SIV_*mac*_ or HIV pathogenesis.

#### Tripartite Motif Proteins 21 and 32 (*TRIM*21 and *TRIM*32)

*TRIM*21 and *TRIM*32 are among the strongest candidate genes for SIV_*mac*_ resistance in Chinese rhesus macaques identified in this study, as they are some of the most divergent orthologs in the entire rhesus macaque genome and are known to play an important role in HIV-1 pathogenesis. These genes are members of a family of tripartite motif (TRIM) proteins that are known to be involved in a variety of cellular functions, including differentiation, apoptosis, and immunity [32]; recently, a number of TRIM proteins have been found to display anti-retroviral activities, and have been associated with innate immunity [32–34]. In our analysis, however, the amino acid sequences corresponding to *TRIM*32, were localized on different chromosomes, and thus have elevated odds of being an ortholog misclassification or being poorly localized in one of the draft sequences.

The exact role of *TRIM*32 in HIV-1 pathogensis is still unclear, as some studies have found that it functions to repress viral gene expression [33], affect trafficking of viral protein [34, 35], or attenuate transcription [36, 37], while others have found that it affected HIV release from cells, but not viral gene expression [38].

*TRIM*32 has been shown to modulate interferon production and cellular antiviral responses by targeting *MITA*/*STING* [39]. In addition, *TRIM*32 plays a key role in *TNF*-*α* induced apoptosis [40].

A human study found significant difference in *TRIM*32 expression between natural elite controllers and anti-retroviral theory (ART) suppressed individuals, with *TRIM*32 expression being elevated in the elite controllers [41]. The comparison between elite controllers and an ART suppressed ‘control’ group is justified by the authors because HIV-1 replication drives expression of many restriction factors (such as TRIM32) and a comparison of gene expression in two ‘aviremic’ yet infected groups (human elite controllers and ART suppressed individuals) minimizes the confounding differences in HIV-1 antigen levels that would be introduced by uninfected controls, or untreated individuals [41]. This study indicates that heightened expression levels of *TRIM*32 might decrease viral replication, perhaps through interactions with HIV-1 Tat [34, 37].

A chimeric protein composed of rhesus macaque-derived *TRIM*5*α* and human-derived *TRIM*21 has been shown to potently inhibit HIV-1 infection [42, 43]. Further, *TRIM*21*CypA*, a *TRIM*21-cyclophilin-A fusion protein, provides highly potent protection against HIV-1 without disturbing normal TRIM activity [44]. Some authors have even suggested that *TRIM*21 itself may be directly involved in anti-HIV activity [45].

More generally, a comparison of human and mouse *TRIM* proteins found that 21% of all mouse, but only 11% of all human *TRIM* proteins interfere with HIV-1 release [38]. We hope to see such comparative methods expanded to include comparison of Chinese and Indian rhesus macaque derived *TRIM* proteins, as *TRIM*21 and *TRIM*32 proteins derived from Chinese rhesus macaques may prove to have especially potent anti-HIV properties relative to those of Indian rhesus macaques.

#### CXC Chemokine Ligand 12 (*CXCL*12)

*CXCL*12 is a natural ligand for the HIV-1 coreceptor CXC Chemokine Receptor 4 (*CXCR*4) [46, 47]. By blocking CXCR4, a key coreceptor for HIV-1 entry, CXCL12 functions as an endogenous inhibitor of some HIV-1 strains [48].

It has been shown that *CXCL*12 is expressed at mucosal surfaces and can strongly reduce the transmission and propagation of X4-HIV at such sites [49], which helps to explain the differential pathogenesis of R5- and X4-SHIV in rhesus macaque models [50]. A naturally occurring splice variant of *CXCL*12 has been shown to be a potent HIV-1 entry inhibitor [51].

It was thought that a SNP (rs1801157) on *CXCL*12 might modulate plasma levels, but this effect was not found [46, 52]. Further, this SNP on *CXCL*12 has been thought to augment risk for HIV-1; an effect has been found in some studies, although the effect size is normally small [46]. A recent meta analysis, however, failed to find any significant association between this SNP and HIV-1 susceptibility [53]. Analysis of genetic variation in *CXCL*12 across Chinese and Indian rhesus macaques, may prove useful in determining if specific variants of *CXCL*12 confer increased resistance to SIV_*mac*_, which might in turn help clarify the role that *CXCL*12 variants might play in HIV-1 pathogenesis in humans.

#### Cyclin-dependent kinase 9 (*CDK*9)

*CDK*9 is a cyclin-dependent kinase that has been linked to HIV-1 pathogenesis in numerous studies—the CDK9/cyclin T1 enzyme, especially, has been shown to play an important role in HIV-1 transcription [54–56]. Due to the central nature of CDK9/cyclin T1 in HIV-1 transcription, several studies have investigated *CDK*9 as a potential drug target [57, 58].

CDK9 is also one of the main host genes that interacts with two HIV genes, *nef* and *tat* [59–62]. Moreover, CDK9 directly affects HIV gene expression and replication [63, 64].

Little is known about the role of *CDK*9 orthologs in SIV_*mac*_ pathogenesis *in vivo* in rhesus macaques, but mutant variants of *CDK*9 lacking kinase activity have been shown to augment the basal transcriptional activity of the HIV-1 long terminal repeat [55].

#### DNA repair protein (*RAD*50)

HIV-1 replication depends on integration of virally produced DNA into the host-cell’s genome via integrase-dependent linkage [65]. It is argued that this linkage leaves an intermediate state that requires post-integration repair; HIV-1 is thought to exploit host double-strand DNA break (DSB) repair pathways in order to complete such repairs [65]. *RAD*50, along with Nijmegen Breakage Syndrome-1 protein (*NBS*1) and Meiotic Recombination 11 Homologue (*MRE*11), is thought to play a role in this process [65]. Little is known about the relationship between *RAD*50 variants and HIV-1 pathogenesis.

#### N-methyl-D-aspartate 1 (*NMDAR*1)

*NMDAR*1 activation is thought to play a role in HIV-1 pathogenesis by modulating HIV-1 gp120-induced blood-brain barrier leakage, in that *NMDAR*1 antagonists have been shown to protect the blood-brain barrier from gp120-induced damage [66]. *NMDAR*1 has been shown to be significantly down regulated in HIV-1 infected astrocytes as opposed to control astrocytes [67].

Little is known about the relationship between *NMDAR*1 variants and HIV-1 pathogenesis; however, a dissertation on differential expression found that the NR1 subunit was significantly downregulated in SIV-infected Indian rhesus macaques, whereas the SIV-infected Chinese rhesus macaques showed no such signs of NR1 downregulation [68].

#### Mucin 16 (*MUC*16)

*MUC*16 is a large membrane-associated mucin [69, 70]. Mucins are present at the surface of epithelial cells in the male and female genital tracts and are thought to play an important role in immune defense by trapping and eliminating pathogens before they can cause infection [70]. Some mucins have been found to be potent in neutralizing HIV-1 (e.g. *MUC*5*B*, *MUC*6, and *MUC*7) [71, 72], but the role of *MUC*16 is yet to be investigated.

#### Other Genes of Interest

*ANKRD*30*A* is a recently-discovered host factor for HIV-1 infection [25]; however, little is known about the role of *ANKRD*30*A* in HIV biology [73].

*IL*13*RA*1 was shown to vary in expression between HIV-1 infected and non-infected controls [74].

Expression of *NT*5*M*, a 5’-nucleotidase gene, was shown to vary (downregulation in macrophages) in response to Tenofovir, a pre-exposure prophylaxis used to prevent HIV-1 infection. Tenofovir functions to cause chain termination when incorporated into viral cDNA, and can thus inhibit viral replication [75]. Although Tenofovir has a direct effect on HIV-1 pathogenesis, immunomodulatory effects of Tenofovir, such as increased expression and secretion of cytokines and modulated expression of 5’-nucleotidases like *NT*5*M* and *NT*5*E*, may also be responsible for its anti-viral activity [75].

The Tat protein of HIV-1 has been shown to interact with *Notch*1 *in vitro* [76]. Further studies have demonstrated linkages between HIV-1 and HIV-associated nephropathy (and progressive renal failure), which is thought to arise through activation of the Notch signaling pathway [77].

*PDCD*5 is a signal gene for apoptosis and was found to be upregulated in HIV-infected individuals relative to controls [78].

*CTSZ* was one of 221 differentially expressed genes up-regulated in the peripheral blood mononuclear cells of HCV/HIV co-infected individuals versus non-infected controls [79].

*GORASP*2 was found to regulate HIV-1 infection, as it was one of 37 genes that decreased MAGI cell *β*-galactosidase reporter activity at least 2-fold for at least three out of four siRNAs in a study of HIV-responsive phosphoproteins [80].

*GTF*2*H*1 was found to be strongly upregulated after monocyte-derived dendritic cells were experimentally infected with HIV-1 [81].

Several SNPs in the region of *TM*9*SF*2 showed signs of association with human AIDS progression *in vivo* [82].

*LPL*, lipoprotein lipase, was associated with HIV in a genome-wide siRNA screen [25]; however, the role that *LPL* plays in HIV pathogenesis, if any, is not well understood. This being said, it has been shown that some HIV protease inhibitors bind to a site on HIV-1 protease that shares approximately 60% homology to a region within *LPL* [83]. For this reason, anti-retroviral therapy has been implicated in peripheral fat wasting (lipodystrophy), central adiposity, hyperlipidaemia, and insulin resistance in human subjects [83].

Knockdown of *ICAM*4 and *BCAR*1 by siRNA enhances the early stages of HIV-1 replication in HIV-infected CD4 cells [22].

No laboratory studies were found that discuss the role of *CYP*26*B*1, *EFR*3*A*, *NME*6, *PIGX*, *BCAR*1, *DSP*, *SLC*35*B*1, *DAPK*2, *PARVA*, or *LYPLA*1 in HIV or SIV pathogenesis; evidence linking these genes to HIV pathogenesis come primarily from genome-wide siRNA analyses [22–25].

### Shortcomings of this Study

Notably, 3 of the 20 orthologs in Table 1, and 4 of the 20 orthologs in Table 2, occurred in different chromosomes. As the evolutionary distance between proposed orthologs increases, it becomes more likely that they may actually be misclassified, especially if the pair breaks synteny. We cannot be sure if these transpositions are real, or if they simply result from classification errors by the RSD algorithm.

Furthermore, this study contains a significant shortcoming in that it uses a form of comparative genomic analysis that is more aptly applied to inter-species comparisons than to intra-species, inter-subspecies comparisons. This is because the use of single genome sequences to estimate the evolutionary divergence of orthologs is more defensible when amino acid sequences are more variable between groups than within groups. This assumption is not necessarily met in our study, because there is a strong potential for many of the amino acid sequences included in this analysis to be as variable within groups as between groups.

There are sure to be numerous amino acid sequences in our data for which our results reduce simply to a comparison between *individual animals*, rather than a true comparison between the normative genomic characteristics of *sub-subspecies*. While this weakness is inherent in our study design, it does not necessarily invalidate our results. Numerous genetic comparisons of Chinese and Indians rhesus macaques have shown that they differ genetically in normative ways at many loci. For instance, of 661 genic SNPs distributed almost uniformly across the genome, 457 were found strictly in one sub-population [84]. Furthermore, discriminant analysis of principal components conducted using 2,808 evenly distributed, (mostly) non-coding SNPs found that the genomic profiles of Chinese rhesus macaques clustered tightly together, with almost no overlap with the distribution of the genomic profiles of Indian rhesus macaques [85]. Further research, however, is needed to investigate amino acid sequence variation in candidate loci across rhesus macaque subspecies using multiple animals from each subspecies.

### Conclusions

We used estimates of the evolutionary distance of orthologous amino acid sequences in Chinese and Indian rhesus macaques to create a candidate list of orthlogs that might be diverging due to selection pressures or demographic processes. To the extent that normative phenotypic differences in immune response to SIV_*mac*_ are due to difference in amino acid sequences across rhesus macaque sub-populations, SIV_*mac*_ resistance in Chinese, but not Indian rhesus macaques should be associated with divergent orthologs. We cross-tabulated a list of divergent orthologs with lists of genes known to be involved in HIV or SIV pathogenesis. We identified 20 candidate genes that occurred on both gene lists and found that several other highly divergent orthologs had been previously linked to HIV or SIV as well.

There appear to have been few or no prior studies of differences in coding variants or expression of these genes between rhesus macaque sub-populations, nor studies investigating the effect of differing rhesus macaque variants of these genes on SIV or HIV pathogenesis. Such studies in the future may help to shed light on the molecular mechanisms underlying differences in normative SIV_*mac*_ resistance between these macaque subspecies.

Comparative genomic work can narrow the set of candidate genes to be investigated with more rigorous laboratory methods and case control studies; careful evaluation of genetic differences in these candidate loci across Indian and Chinese rhesus macaque sub-populations, and evaluation of the effect of these differences on SIV_*mac*_ pathogenesis, is needed in order to identify the structural and functional differences in genes underpinning SIV_*mac*_ resistance in Chinese, but not Indian rhesus macaques.

## Materials and Methods

### Data Sources

Amino acid sequence data for the Chinese rhesus macaque was accessed from the Comprehensive Library for Modern Biotechnology, BGI <http://climb.genomics.cn/10.5524/100002>, and amino acid sequence data for the Indian rhesus macaque was accessed from the Monkey Database, BGI <http://macaque.genomics.org.cn/page/species/index.jsp> in February, 2014.

### Software, Programming Environment, and Analytical Methods

A Python implementation of the RSD 1.1.6 algorithm [15] was used in data analysis. Local installation of the script was conducted following instructions on GitHub <https://github.com/todddeluca/reciprocal_smallest_distance>. RSD 1.1.6 depends on Python 2.7, NCBI BLAST 2.2.26, PAML 4.4, and Kalign 2.04, so these programs were installed locally as well (February, 2014).

The GOanna [19] program was used to associate amino acid sequence data with human gene IDs and gene ontology annotations.

All other data analysis and visualization was conducted in the the R programming environment [86]; the *seqinr* package was used to read the FASTA sequence files.

Statistical modeling was conducted using the Stan version 2.5 C++ library for Hamiltonian Markov Chain Monte Carlo simulation, using the *RStan* package in R [87].

### Statistical Methods

We use a multi-level zero-inflated gamma regression model to estimate differences in the evolutionary distance of orthologs as a function of chromosome and chromosomal position. Random effects on chromosome control for heterogeneity in evolutionary distance across chromosomes, and chromosome-specific vectors of random effects generated using a Gaussian process control for heterogeneity in evolutionary rate as a function of location on each chromosome. More formally, on each chromosome, *c*, we model the evolutionary distance of the *n* ^*th*^ amino acid sequence, *Y*_[*c*,*n*]_, using the probability function:

$$\begin{array}{c} Y_{\left[c,n\right]}∼\{\begin{array}{cc} \text{Gamma}(A_{\left[c,n\right]},B_{\left[c\right]}) & \;\text{if}\;Y_{\left[c,n\right]}>0,\;\text{and}\\ \text{Bernoulli}(\theta_{\left[c,n\right]}) & \;\text{if}\;Y_{\left[c,n\right]}=0 \end{array} \end{array}$$ (1)

We note that the mean of a gamma distribution is defined as $\mu =\frac{A}{B}$. As such, *A*_[*c*,*n*]_ is defined from a model of the mean of a gamma distribution as:

$$\begin{array}{c} A_{\left[c,n\right]}=\text{exp}\left(\alpha_{\left[c\right]}+\gamma_{\left[c\right]}\delta_{\left[c,Z(n)\right]}\right)B_{\left[c\right]} \end{array}$$ (2)

and *θ*_[*c*,*n*]_ is defined through a similar link function:

$$\begin{array}{c} \theta_{\left[c,n\right]}=\text{logistic}\left(\beta_{\left[c\right]}+\nu_{\left[c\right]}\Phi_{\left[c,Z(n)\right]}\right) \end{array}$$ (3)

The parameters *α*_[*c*]_ and *β*_[*c*]_ ∈ ℝ are chromosome specific intercepts. The parameters *γ*_[*c*]_ and *ν*_[*c*]_ ∈ ℝ^+^ scale the variance of the random effects, *δ*_[*c*,*Z*(*n*)]_ and *ϕ*_[*c*,*Z*(*n*)]_. The vectors *δ*_[*c*,1,…,*Z*]_ and *ϕ*_[*c*,1,…,*Z*]_ (where *Z* = 40 is the number of equally-sized zones modeled per chromosome) are estimated using mean zero Gaussian processes:

$$\begin{array}{c} \delta_{\left[c,1,\ldots ,Z\right]}∼\text{Multivariate}\;\text{Normal}\left({\left(0,\ldots ,0\right)}^{\prime },\Gamma \right) \end{array}$$ (4)

$$\begin{array}{c} \phi_{\left[c,1,\ldots ,Z\right]}∼\text{Multivariate}\;\text{Normal}\left({\left(0,\ldots ,0\right)}^{\prime },\Pi \right) \end{array}$$ (5)

where the correlation matrices Γ and Π are formed as functions of the unit-normalized distance, *D*, between chromosomal zones *z*_1_ and *z*_2_ squared:

$$\begin{array}{c} \Gamma_{\left[z_{1},z_{2}\right]}=\{\begin{array}{cc} \rho_{\left[c\right]}\;\text{exp}\left(-\zeta_{\left[c\right]}D_{\left[z_{1},z_{2}\right]}^{2}\right) & \;\text{if}\;z_{1}\neq z_{2},\;\text{and}\\ 1 & \;\text{if}\;z_{1}=z_{2} \end{array} \end{array}$$ (6)

$$\begin{array}{c} \Pi_{\left[z_{1},z_{2}\right]}=\{\begin{array}{cc} \kappa_{\left[c\right]}\;\text{exp}\left(-\xi_{\left[c\right]}D_{\left[z_{1},z_{2}\right]}^{2}\right) & \;\text{if}\;z_{1}\neq z_{2},\;\text{and}\\ 1 & \;\text{if}\;z_{1}=z_{2} \end{array} \end{array}$$ (7)

where the parameters *ρ* and *κ* ∈ (0, 1) represent the maximum correlation, and the parameters *ζ* and *ξ* ∈ ℝ^+^ modulate the decay of correlation with distance between chromosomal zones. A more finely resolved Gaussian process would use a random effect on each observation, but given that the computational requirements of a Gaussian process model scale at about O(*n* ^3^), it is unfeasible to model each data point as arising from a Gaussian process. Instead, we divide each chromosome into *Z* = 40 ordered and equally dense zones, and let the Gaussian process generate a random effect that is shared among all amino acid sequences in that zone. We denote the zone of the *n* ^*th*^ amino acid sequence on a chromosome using the function notation *Z*(*n*), to return the zone, *z*, of the *n* ^*th*^ amino acid sequence.

To complete the Bayesian model, we use multi-level partial pooling to share information across chromosomes:

$$\begin{array}{c} \alpha_{\left[c\right]}∼\text{Normal}\left(\mu_{\alpha },\sigma_{\alpha }\right) \end{array}$$ (8)

$$\begin{array}{c} \beta_{\left[c\right]}∼\text{Normal}\left(\mu_{\beta },\sigma_{\beta }\right) \end{array}$$ (9)

We specify weakly regularizing priors on *μ*_*α*_ and *μ*_*β*_:

$$\begin{array}{c} \mu_{\alpha }∼\text{Normal}\left(0,10\right) \end{array}$$ (10)

$$\begin{array}{c} \mu_{\beta }∼\text{Normal}\left(0,10\right) \end{array}$$ (11)

and vague priors on *σ*_*α*_ and *σ*_*β*_:

$$\begin{array}{c} \sigma_{\alpha }∼\text{Cauchy}\left(0,3\right)T\left[0,\infty \right] \end{array}$$ (12)

$$\begin{array}{c} \sigma_{\beta }∼\text{Cauchy}\left(0,3\right)T\left[0,\infty \right] \end{array}$$ (13)

where the truncation operator, *T*[0, ∞], indicates truncated support on the interval (0, ∞). Each element of the vector of scale parameters, *B*, for the gamma regression model is given a weakly informative prior:

$$\begin{array}{c} B_{\left[c\right]}∼\text{Normal}\left(0,10\right)T\left[0,\infty \right] \end{array}$$ (14)

Each element of *γ* and *ν*, the variance parameters, get vague priors:

$$\begin{array}{c} \gamma_{\left[c\right]}∼\text{Cauchy}\left(0,3\right)T\left[0,\infty \right] \end{array}$$ (15)

$$\begin{array}{c} \nu_{\left[c\right]}∼\text{Cauchy}\left(0,3\right)T\left[0,\infty \right] \end{array}$$ (16)

Because the elements of *ρ* and *κ* are constrained to the unit interval, they get weakly informative Beta priors:

$$\begin{array}{c} \rho_{\left[c\right]}∼\text{Beta}\left(2,2\right) \end{array}$$ (17)

$$\begin{array}{c} \kappa_{\left[c\right]}∼\text{Beta}\left(2,2\right) \end{array}$$ (18)

Finally, the elements of the decay parameter vectors, *ζ* and *ξ*, get weakly informative priors:

$$\begin{array}{c} \zeta_{\left[c\right]}∼\text{Normal}\left(2,3\right)T\left[0,\infty \right] \end{array}$$ (19)

$$\begin{array}{c} \xi_{\left[c\right]}∼\text{Normal}\left(2,3\right)T\left[0,\infty \right] \end{array}$$ (20)

## Supporting Information

S1 Table This file (Excel format) contains the full set of orthologs and evolutionary distances from our analysis. We have marked the genes that break synteny.
(XLSX)

S2 Table This file (Excel format) contains the full set of previously described HIV-related genes utilized to construct results presented in Table 1. (XLSX)

S3 Table This file (Excel format) contains the full set of gene function classifications for which GOanna could amend annotations and for which the RSD algorithm found orthologs. (XLSX)

S4 Table This file (Excel format) contains the summary of the posterior parameter estimates from MCMC simulation using the Stan 2.5 C++ library. (XLSX)

S1 Code This file (R script) contains the code used to construct our statistical model. (R)
